# Comparative Analysis of the Roles of Non-muscle Myosin-IIs in Cytokinesis in Budding Yeast, Fission Yeast, and Mammalian Cells

**DOI:** 10.3389/fcell.2020.593400

**Published:** 2020-11-19

**Authors:** Kangji Wang, Hiroki Okada, Erfei Bi

**Affiliations:** Department of Cell and Developmental Biology, Perelman School of Medicine, University of Pennsylvania, Philadelphia, PA, United States

**Keywords:** Myo1, Myo2, Myp2, non-muscle myosin-II, cytokinesis, budding yeast, fission yeast, mammalian cells

## Abstract

The contractile ring, which plays critical roles in cytokinesis in fungal and animal cells, has fascinated biologists for decades. However, the basic question of how the non-muscle myosin-II and actin filaments are assembled into a ring structure to drive cytokinesis remains poorly understood. It is even more mysterious why and how the budding yeast *Saccharomyces cerevisiae*, the fission yeast *Schizosaccharomyces pombe*, and humans construct the ring structure with one, two, and three myosin-II isoforms, respectively. Here, we provide a comparative analysis of the roles of the non-muscle myosin-IIs in cytokinesis in these three model systems, with the goal of defining the common and unique features and highlighting the major questions regarding this family of proteins.

## Introduction

Non-muscle myosin-IIs (NM-IIs) play critical roles in many fundamental processes including cytokinesis, cell adhesion, cell migration, exocytosis, and tissue morphogenesis (Vicente-Manzanares et al., 2009; Shutova and Svitkina, 2018). Interestingly, the budding yeast *Saccharomyces cerevisiae*, the fission yeast *Schizosaccharomyces pombe*, and humans possess one, two, and three isoforms of the NM-IIs, respectively (Watts et al., 1987; Bezanilla et al., 1997; Kitayama et al., 1997; May et al., 1997; Motegi et al., 1997; Bi et al., 1998; Lippincott and Li, 1998; Golomb et al., 2004). While the *in vitro* assembly of the mammalian NM-IIs and its regulation by phosphorylation have been studied and reviewed extensively (Trybus, 1991; Tan et al., 1992; Ronen and Ravid, 2009; Vicente-Manzanares et al., 2009; Shutova and Svitkina, 2018), the *in vivo* assembly, architecture, function, and regulation of these NM-IIs are not well understood. Much less is known about myosin-II assembly or its lack thereof in both yeasts. Here, we compare and contrast the roles of the NM-IIs in cytokinesis in budding yeast, fission yeast, and humans to define the commonalities and differences for this important family of proteins.

Cytokinesis in fungal and animal cells requires concerted actions of an actomyosin ring (AMR), targeted vesicle fusion, and localized ECM remodeling (Balasubramanian et al., 2004; Pollard and Wu, 2010; Meitinger and Palani, 2016; Bhavsar-Jog and Bi, 2017; Pollard and O’Shaughnessy, 2019). The AMR consists of NM-IIs and actin filaments and is thought to produce a contractile force that drives cleavage furrow ingression. In both budding and fission yeast, the AMR also guides exocytosis and localized cell wall synthesis (equivalent of ECM remodeling in animal cells) (Vallen et al., 2000; Schmidt et al., 2002; Fang et al., 2010; Proctor et al., 2012; Thiyagarajan et al., 2015; Palani et al., 2017; Okada et al., 2019). Reciprocally, the newly synthesized ECM at the division site stabilizes the AMR (Bi, 2001; Schmidt et al., 2002; Verplank and Li, 2005). Whether a similar AMR–ECM relationship exists in mammalian cells remains unknown. It is also a central mystery as to why and how cytokinesis is driven by one NM-II (defined by the heavy chain gene *MYO1*) in budding yeast, two (*MYO2* and *MYP2*) in fission yeast, and three (*MYH9*, *MYH10*, and *MYH14*) in mammalian cells. Here, we attempt to shed some light on this mystery by comparative analysis of the roles of the NM-IIs in cytokinesis in these diverse model systems.

## Myo1: The Sole Myosin-II Heavy Chain in Budding Yeast

**FIGURE 1:**
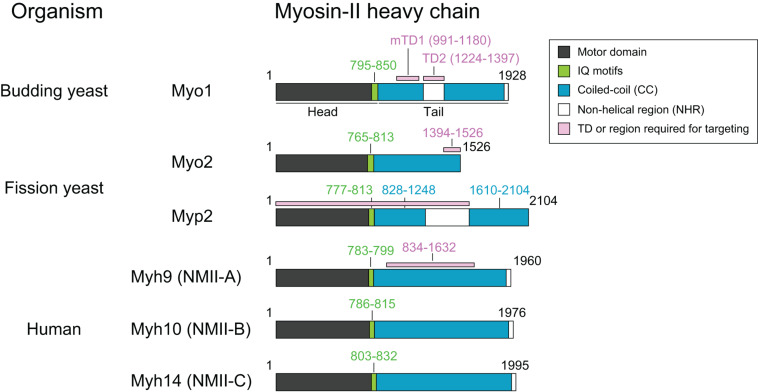
Common features of myosin-II isoforms in *S. cerevisiae*, *S. pombe*, and *H. sapiens*. Motor, motor domain with ATPase and actin-binding sites; IQ, IQ1, and IQ2 motifs—the binding sites for the essential light chain (ELC) and regulatory light chain (RLC), respectively. CC, coiled-coil region; NHR, non-helical region; TD, targeting domain, which is defined as the region of a protein that is both necessary and sufficient for its localization to the division site.

The budding yeast *S. cerevisiae* has only one myosin-II heavy chain Myo1 (a misnomer for a historical reason) (Figure 1), one essential light chain (ELC) Mlc1, and one regulatory light chain (RLC) Mlc2 (Luo et al., 2004). Mlc1 is also a light chain for the myosin-V Myo2 as well as for the sole IQGAP Iqg1 in budding yeast (Stevens and Davis, 1998; Boyne et al., 2000; Shannon and Li, 2000; Luo et al., 2004). Deletion of *MYO1* causes pronounced defects in cytokinesis and cell separation but not cell lethality in most strain backgrounds (Watts et al., 1987; Rodriguez and Paterson, 1990; Bi et al., 1998; Lippincott and Li, 1998). Thus, the budding yeast is ideally suited for dissecting the structure–function relationship of a NM-II, especially in the context of cytokinesis.

### The Structure and Assembly of Myo1

Myo1 consists of 1,928 amino acids, with a predicted globular head (aa1–855) containing an actin-binding site and ATPase domain, two IQ motifs (aa795–850) where the ELC and RLC bind, and a coiled-coil (CC) tail (aa856–1,928) (Figure 1; Luo et al., 2004). Remarkably, deletion of the IQ1 and IQ2 motifs does not cause any obvious defects in cytokinesis (Luo et al., 2004). Myo1 tail is also predicted to contain two non-helical regions (NHRs), an internal NHR (residues 1,224–1,397) that contains seven helix-breaking proline residues and a C-terminal NHR (residues 1,914–1,928) (Fang et al., 2010; Wloka et al., 2013). Studies of Myo1 purified from yeast by rotary-shadowing electron microscopy (EM) indicate that Myo1 forms a two-headed structure with a rod tail, similar to the NM-IIs in other organisms (Fang et al., 2010). The Myo1 tail forms a kink at the position corresponding to the internal NHR (Fang et al., 2010). Analysis of the native septin architectures at the division site during the cell cycle by platinum-replica EM (PREM) suggests that Myo1 forms filaments during cytokinesis (Ong et al., 2014; Chen et al., 2020), although it remains unknown whether Myo1 forms bipolar filaments *in vitro* without the assistance of some accessary factors.

### Localization and Dynamics of Myo1 During the Cell Cycle

**FIGURE 2:**
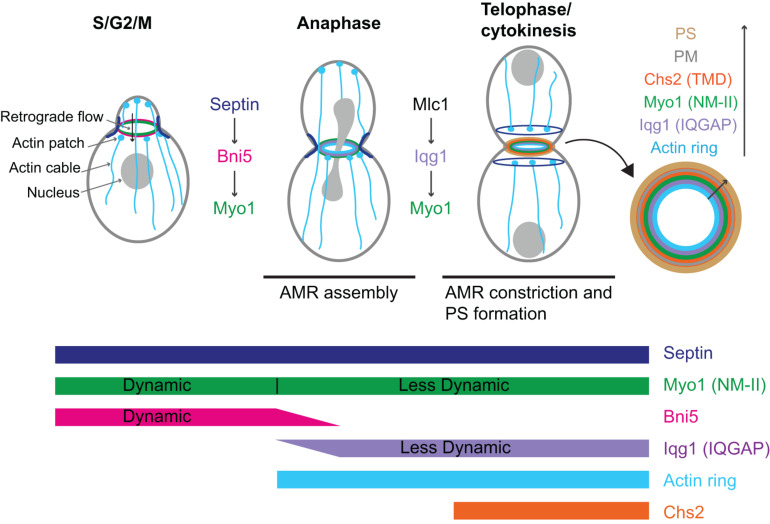
Localization, dynamics, function, and regulation of Myo1 during the cell cycle in budding yeast. AMR, actomyosin ring; PS, primary septum; PM, plasma membrane; Chs2 (TMD), chitin synthase-II (transmembrane domain protein). See text for details.

Myo1 localizes to the division site in a biphasic pattern (Fang et al., 2010; Figure 2). Before anaphase, Myo1 is recruited to the division site by the septin-binding protein Bni5 (Figure 2; Fang et al., 2010). Bni5 binds to both the minimal targeting domain 1 (mTD1, aa991–1,180) in the Myo1 tail (Figure 1) and the C-terminal tails of the septins Cdc11 and Shs1 (Lee et al., 2002; Fang et al., 2010; Finnigan et al., 2015). The mTD1 is necessary and sufficient for Myo1 localization to the division site before anaphase (Fang et al., 2010). During telophase or cytokinesis, Myo1 is maintained at the division site by Iqg1 (Fang et al., 2010), the sole and essential IQGAP in budding yeast (Figure 2; Epp and Chant, 1997; Lippincott and Li, 1998). As the neck localization of Iqg1 depends on Mlc1 (Boyne et al., 2000; Shannon and Li, 2000), not surprisingly, the maintenance of Myo1 at the division site during cytokinesis also depends on Mlc1 (Figure 2; Fang et al., 2010). Strikingly, the targeting domain 2 (TD2, aa1,224–1,397) in the Myo1 tail, which is essentially the internal NHR, is necessary and sufficient for Myo1 localization at the division site during cytokinesis (Figure 1; Fang et al., 2010). While the localization dependency is clear, no direct interaction between Myo1 or its TD2 and Iqg1 has been detected (Fang et al., 2010). The Bni5- and Iqg1-mediated mechanisms for Myo1 targeting presumably overlap during anaphase, with the Bni5 mechanism dampening and the Iqg1 mechanism strengthening (Fang et al., 2010; Figure 2). The switch between the two mechanisms is regulated largely at the level of Bni5 degradation and Iqg1 expression during the cell cycle (Epp and Chant, 1997; Lippincott and Li, 1998; Lee et al., 2002).

Because Mlc1, the ELC for Myo1 (Luo et al., 2004), is also a light chain for Myo2 and Iqg1 (Stevens and Davis, 1998; Boyne et al., 2000; Shannon and Li, 2000), its localization mechanism appears more complex. The localization of Mlc1 to the division site before cytokinesis depends on the septin hourglass (Boyne et al., 2000; Shannon and Li, 2000; Luo et al., 2004) and this targeting mechanism is chiefly mediated by the binding of Mlc1 to Myo1 (Feng et al., 2015). The maintenance of Mlc1 at the division site during cytokinesis depends on filamentous actin (F-actin) and the formin Bni1 (Feng et al., 2015), which localizes to the division site during cytokinesis (Pruyne et al., 2004; Buttery et al., 2007). Mlc1 fails to localize to the division site when the septin structure and F-actin are simultaneously disrupted (Feng et al., 2015). In contrast, the localization of the RLC Mlc2 to the division site completely depends on its binding to Myo1 (Luo et al., 2004).

Fluorescence recovery after photo-bleaching (FRAP) analysis indicates that prior to anaphase, there is little or no exchange of Myo1 between the division site and the cytosol, suggesting that nearly all the Myo1 molecules are localized to the division site. However, Myo1 is highly dynamic within the division site (Dobbelaere and Barral, 2004; Wloka et al., 2013; Figure 2). This dynamic pattern of Myo1 is similar to that of its binding partner and recruiter Bni5, suggesting that the dynamics of Myo1, in addition to its localization, at this stage of the cell cycle is likely regulated by Bni5 (Wloka et al., 2013). Interestingly, after the onset of anaphase, Myo1 is progressively immobilized, going from a partially “frozen” state during anaphase to a completely frozen state during cytokinesis (Figure 2; Dobbelaere and Barral, 2004; Wloka et al., 2013). Not surprisingly, Mlc1 and Iqg1, the stabilizers of Myo1 at the division site during this stage of the cell cycle, begin to localize to the division site after anaphase and are frozen during their entire durations at the division site (Wloka et al., 2013). Remarkably, Mlc1 and Iqg1 become dynamic in the absence of Myo1, suggesting that the immobility of Myo1 is foundational to the immobility of other cytokinetic proteins (Wloka et al., 2013). These observations also suggest that Mlc1/Iqg1 and Myo1 regulate each other during cytokinesis, with Mlc1/Iqg1 being required for Myo1 localization while Myo1 being required for Mlc1/Iqg1 organization or turnover. In contrast to the ELC Mlc1, the RLC Mlc2 displays a similar change in dynamics during the cell cycle as Myo1 does (Wloka et al., 2013). This is not surprising, as Mlc2 binds to Myo1 and this binding is essential for its localization to the division site throughout the cell cycle (Luo et al., 2004). Further analysis indicates that the head domain of Myo1 is not required for its frozen state (Wloka et al., 2013). However, a small truncation at its C-terminus [e.g., Myo1-(aa1–1,798)] makes Myo1 at this stage dynamic again (Wloka et al., 2013). Collectively, these observations suggest that Myo1 might form a highly ordered structure during cytokinesis and the tail region near its C-terminal end is required for this assembly (Wloka et al., 2013). Alternatively, the C-terminal truncations may not abolish Myo1 assembly, but might make the assembled structures more mobile at the division site, perhaps due to a weakened C-terminus-mediated association with the plasma membrane (PM). As five distinct C-terminal truncations of Myo1, including Myo1-(aa1–1,798), were isolated from the synthetic-lethal screen with the deletion of *HOF1*, which encodes an F-BAR protein involved in cytokinesis, the C-terminal region of Myo1 must be important for its function (Nishihama et al., 2009; Wloka et al., 2013).

### The Functions of Myo1 in Cytokinesis and Beyond

Myo1 localizes to the presumptive bud site and then to the bud neck from bud emergence to the completion of cytokinesis (i.e., membrane closure between the mother and daughter cells). However, actin filaments join Myo1 at the bud neck to form an AMR only after the onset of anaphase. Thus, the function of Myo1 during the cell cycle can be divided into two stages: before and after anaphase.

#### The Function of Myo1 in Retrograde Flow of Actin Cables Before Anaphase

Myo1 facilitates the retrograde flow of actin cables before anaphase (Figure 2; Huckaba et al., 2006). Retrograde flow is a process conserved from yeast to mammalian cells. In mammalian cells, actin retrograde flow promotes receptor recycling and cell migration, and this process requires actin polymerization and myosin-II activity (Lin et al., 1997; Yang et al., 2012; Yi et al., 2012; Swaminathan et al., 2017). In budding yeast, the actin cables nucleated by the formin Bni1 at the bud cortex undergo retrograde flow that is involved in the inward movement of actin patches, the sites of endocytosis, and endosomes as well as in mitochondrial inheritance (Fehrenbacher et al., 2004; Huckaba et al., 2004). The same actin cables are also required for the anterograde transport of various cargoes including secretory vesicles, mRNAs, vacuoles, and nucleus that are powered by myosin-Vs (Bretscher, 2003). Thus, actin cables are engaged in bidirectional transport in budding yeast. Myo1 at the bud neck binds to actin cables via its motor domain, which generates a pulling force to increase the rate of the retrograde flow by twofold (Huckaba et al., 2006). This function requires the motor activity as well as the bud-neck localization of Myo1 (Huckaba et al., 2006). By anchoring to the septin hourglass, a diffusion barrier at the bud neck, and moving on the actin cables, Myo1 might help cargoes go through the septin barrier and the narrow bud neck more efficiently (Huckaba et al., 2006). As Bni5 is the linker between Myo1 and the septin hourglass, it is also expected to function in the retrograde flow of actin cables (Fang et al., 2010), a possibility that should be investigated in the future.

#### The Function of Myo1 in Cytokinesis After Anaphase

In budding yeast, AMR constriction is closely followed by the centripetal growth of a primary septum (PS) that is catalyzed by the chitin synthase II (Chs2) (Figure 2; Fang et al., 2010). These two processes are interdependent and act in concert to drive efficient cleavage-furrow ingression (Schmidt et al., 2002). The AMR guides PS formation whereas the PS stabilizes the AMR during its constriction (Vallen et al., 2000; Bi, 2001; Schmidt et al., 2002; Verplank and Li, 2005). In this context, Myo1 plays both motor-dependent and-independent roles in cytokinesis. The head domain of Myo1, including the binding sites for the ELC and RLC, accounts for 25–30% of the constriction rate of the AMR (Lord et al., 2005; Fang et al., 2010). When the head domain is deleted, the Myo1 tail is able to direct the assembly of a “headless AMR” that can largely accomplish cytokinesis by guiding the PS formation (Fang et al., 2010). How the Myo1 tail interacts with actin filaments to assemble the headless AMR remains unknown. It is also unknown whether and how the headless AMR drives furrow ingression through cell-cycle triggered disassembly of Myo1 tail and actin filaments or Chs2-mediated PS synthesis or both.

### Major Unanswered Questions Regarding Myo1 in Budding Yeast

FRAP analysis suggests that Myo1 is organized into a stable structure during cytokinesis (Dobbelaere and Barral, 2004; Wloka et al., 2013). Indeed, PREM analysis suggests that Myo1 might form filaments in the middle region of a transitional septin hourglass at the onset of cytokinesis (Ong et al., 2014; Chen et al., 2020). However, the precise architecture of the AMR, especially the pattern of Myo1 organization, before and during AMR constriction remains unknown. It is also unknown whether Myo1 can form any kind of filaments *in vitro* and whether its filament assembly *in vivo* is regulated by cell cycle-controlled phosphorylation and/or by trans-acting factors such as other cytokinetic proteins. Finally, it remains unclear how Myo1 is spatiotemporally coupled to polarized exocytosis and Chs2-mediated PS formation during cytokinesis.

## Myo2 and Myp2: The Two Myosin-II Heavy Chains in Fission Yeast

The rod-shaped fission yeast, *S. pombe*, evolutionally diverged from its fungal relative, the budding yeast *S. cerevisiae*, ∼420 million years ago (Sipiczki, 2000). Similar to that in budding yeast, cytokinesis in fission yeast requires spatiotemporal coupling of AMR constriction with PS formation (Balasubramanian et al., 2004; Pollard and Wu, 2010; Willet et al., 2015; Zhou et al., 2015; Okada et al., 2019). In contrast, however, *S. pombe* has two myosin-II heavy chains, the major isoform Myo2 (Kitayama et al., 1997; May et al., 1997) and the minor isoform Myp2/Myo3 (hereafter Myp2) (Figure 1; Bezanilla et al., 1997; Motegi et al., 1997). Both Myo2 and Myp2 share the ELC Cdc4 and the RLC Rlc1, and both localize to the division site (Mccollum et al., 1995; Le Goff et al., 2000; Naqvi et al., 2000; D’souza et al., 2001). Similar to budding yeast, Cdc4 in fission yeast is also a light chain for the IQGAP Rng2 and myosin-V Myo51 (D’souza et al., 2001). Myo2 is indispensable for viability and cytokinesis, while Myp2 is only required for survival under stressful conditions such as high salt (Bezanilla et al., 1997; Kitayama et al., 1997; May et al., 1997; Motegi et al., 1997). Thus, the fission yeast is an ideal model system for dissecting the differentiated roles of different myosin-II isoforms in cytokinesis.

### The Structure and Assembly of Myo2 and Myp2

Myo2 and Myp2 share the same basic domains as myosin-IIs in other organisms: a head domain harboring ATPase activity and an actin-binding site, two IQ motifs that bind to ELC and RLC, and a tail domain that is made of CCs, with a NHR in the middle of Myp2 tail (Figure 1; Bezanilla et al., 1997; Kitayama et al., 1997; May et al., 1997; Motegi et al., 1997; D’souza et al., 2001). Similar to Myo1 in budding yeast (Luo et al., 2004), deletion of the IQ1 and IQ2 motifs in Myo2 abolishes the ELC (Cdc4) and RLC (Rlc1) binding, but, surprisingly, does not cause any obvious defects in cytokinesis, even in the absence of Myp2 (D’souza et al., 2001). Analysis of Myo2 and Myp2 chimera indicates that the tail domain determines the protein-specific regulation and function (Bezanilla and Pollard, 2000; Lord et al., 2005).

**FIGURE 3:**
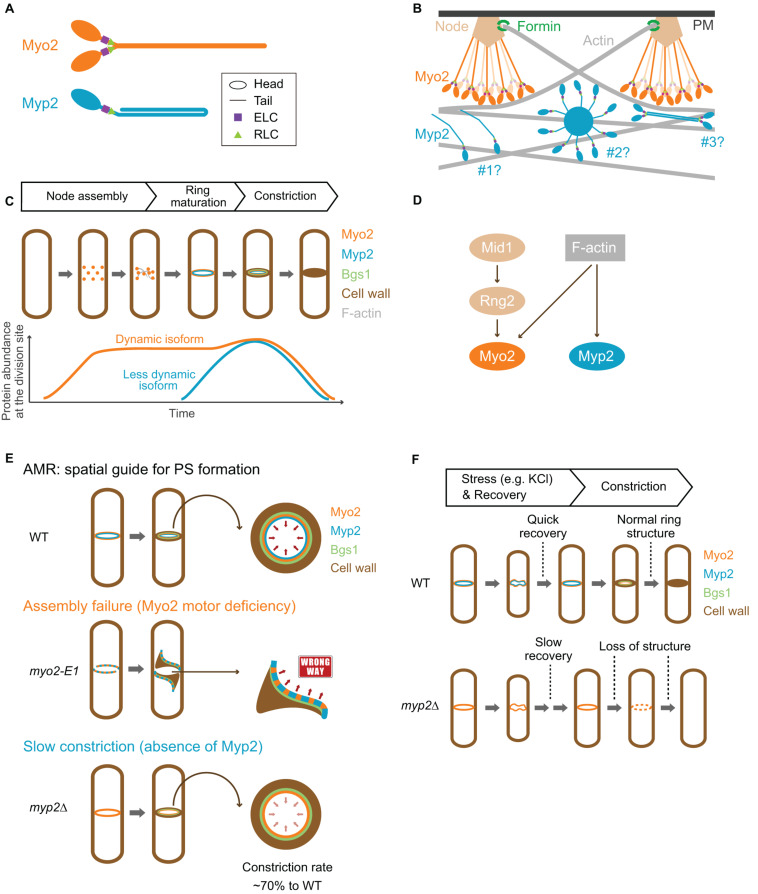
Structure, assembly, localization, dynamics, and function of Myo2 and Myp2 during the cell cycle in fission yeast. **(A)** Molecular structure of Myo2 and Myp2. **(B)** Assembly of Myo2 and Myp2. Modified from Laporte et al. (2011); Takaine et al. (2015), Laplante et al. (2016), and Nguyen et al. (2018). **(C)** Localization of Myo2 and Myp2 during the cell cycle. **(D)** Targeting mechanisms of Myo2 and Myp2. **(E)** The role of the AMR in guiding PS formation. Modified from Okada et al. (2019). **(F)** Stress response of the AMR. See text for details.

Myo2 has a short tail with 711 residues, which is predicted to contain α-helical rod followed by a flexible, less-ordered region due to the presence of proline residues within the last ∼150 residues (Figures 1, 3A). This was validated by rotary-shadowing EM showing that Myo2 forms a two-headed structure with an 85–90 nm rod tail (Bezanilla and Pollard, 2000; Pollard et al., 2017; Friend et al., 2018). In contrast to myosin-IIs in animals and amoebas, purified Myo2 does not form filaments or mini-filaments under a wide range of salt concentrations, including physiological concentration (Pollard et al., 2017; Friend et al., 2018). This is consistent with its unipolar organization in the cytokinesis nodes, as revealed by quantitative high-speed fluorescence photoactivation localization microscopy (FPALM) (Figure 3B; Laplante et al., 2016). In addition, cytosolic Myo2 in interphase cells also exists as a non-filament form (Friend et al., 2018).

Myp2 has a long tail with 1,336 residues, which contains two CC regions separated by a medial proline-rich NHR (Figures 1, 3A). Recombinant Myp2 tail sediments as a monomer and the tail length is 60 nm, which is about half of the length estimated by the residues (∼130 nm), suggesting that Myp2 is a single-headed myosin-II with its tail folded back to form a short rod consisting of antiparallel CCs (Bezanilla and Pollard, 2000). This would essentially place the C-terminal end of Myp2 next to its head domain. However, a recent study by super-resolution microscopy indicates that N- and C-terminal ends of Myp2 are ∼90 nm apart in the assembled AMR (Figure 3B; Mcdonald et al., 2017), suggesting that Myp2 unlikely exists as a single-headed myosin with a folded-back tail *in vivo*. Interestingly, the tail of Myo1 in *S. cerevisiae* has a similar domain structure (two CC regions separated by a proline-rich NHR) (Figure 1). However, Myo1 forms a two-headed structure with “kink” at the proline region (Fang et al., 2010). Moreover, Myo1 appears to form filaments at the division site (Ong et al., 2014; Chen et al., 2020). It is tempting to speculate that Myp2 might also exist as a two-headed monomer that assembles into bipolar filament as proposed in a simulated model of *S. pombe* cytokinesis (Figure 3B; Nguyen et al., 2018).

The assemblies and spatial organization patterns of Myo2 and Myp2 within the contractile ring are distinct (Figure 3B). There is a total of ∼5,000 myosin-II molecules at the division site (3,000 of Myo2 and 2,000 of Myp2) (Wu and Pollard, 2005). Myo2 is thought to assemble into an AMR by a search, capture, pull, and release (SCPR) mechanism, which drives the coalescence of the cytokinetic nodes into a ring structure during metaphase and anaphase A (Vavylonis et al., 2008). After the onset of mitosis, Myo2 is incorporated into the nodes by forming a bouquet of Myo2 molecules (Figure 3B), with their head domains exposed to the cytoplasm and their tails anchored to the PM via the anillin-like protein Mid1, the central organizer of the nodes (Laporte et al., 2011; Laplante et al., 2016; Mcdonald et al., 2017). The Myo2 tails are also associated with several other key cytokinetic proteins including the IQGAP Rng2, the F-BAR protein Cdc15, and the formin Cdc12 in the nodes (Laporte et al., 2011; Laplante et al., 2016). Cdc12 nucleates the assembly of linear actin filaments, which will be captured and pulled by the Myo2 heads in the nearby nodes, resulting in the coalescence of the nodes into a ring structure (Vavylonis et al., 2008). The pulling force inhibits the activity of Cdc12, preventing node entanglement by an excess of actin filaments (Zimmermann et al., 2017). According to the SCPR model (Vavylonis et al., 2008), both the ATPase and the actin-binding activities of Myo2 should be essential for AMR assembly, and, presumably, for constriction as well.

While the assembly of Myo2 in the cytokinetic nodes or ring is best understood among all the NM-IIs, the assembly of Myp2 is far from clear. Disruption of F-actin by Latrunculin A (LatA) abolishes Myp2, but not Myo2, localization, suggesting that Myp2 localizes to and maintains at the division site by binding to actin filaments or other proteins whose localizations depend on F-actin (Wu et al., 2003). This is further corroborated by recent nanoscale analysis of Myp2 in the pre-constricted AMR (Mcdonald et al., 2017). The head domain of Myp2 lies within the F-actin network, ∼210 nm deep from the PM, while the C-terminal tail lies ∼125 nm away from the PM (Figure 3B, scenario #1; Mcdonald et al., 2017). Myp2 was also proposed to form clusters with a kernel that is assembled by Myp2 tails. These clusters could presumably interact with actin filaments persistently, therefore, contributing to the consolidation of and stable association with the actin ring (Figure 3B, scenario #2; Takaine et al., 2015). However, the uniform distribution of Myp2 in the pre-constricted AMR argues against this possibility (Mcdonald et al., 2017). Alternatively, Myp2 might form bipolar filaments, as suggested by computational modeling (Nguyen et al., 2018), which can explain the previous observation that F-actin bundles containing Myp2 are peeled off from the main ring during constriction (Figure 3B, scenario #3; Laplante et al., 2015). However, this possibility does not easily reconcile with the observed distance (∼90 nm) between the N- and C-terminal ends of Myp2 in relation to the PM (Mcdonald et al., 2017). Regardless of the scenarios, overexpression or deletion of the tail region disrupts the Myp2 function (Bezanilla and Pollard, 2000; Takaine et al., 2015; Okada et al., 2019), suggesting that the tail is critical for Myp2 assembly and function.

### Localization and Dynamics of Myo2 and Myp2 During the Cell Cycle

Both Myo2 and Myp2 in *S. pombe* localize to the division site during mitosis but at distinct times (Figure 3C). Upon mitotic entry, Myo2 localizes to the cell equator as a band of nodes, which are membrane-associated precursors of the contractile ring (Wu et al., 2003, 2006). These nodes are subsequently coalesced into a ring during metaphase and anaphase A (Bezanilla et al., 2000; Motegi et al., 2000; Wu et al., 2003; Vavylonis et al., 2008). At the onset of cytokinesis in anaphase B, long before the spindle breakage (Cortes et al., 2018), the Myo2 ring starts to constrict slowly, and then switches to a fast-phase constriction after the spindle breakage or the onset of telophase (Okada et al., 2019). Structural and functional analysis indicates that a 133-aa C-terminal fragment (residues 1,394–1,526) of Myo2 serves as a TD that is necessary and sufficient for its localization to the division site (Figure 1; Motegi et al., 2004). In interphase cells, Myo2 exists in the cytosol (Kitayama et al., 1997; Bezanilla et al., 2000). It was proposed that, upon entry into mitosis, Myo2 is dephosphorylated at Ser1444 within its localization domain, which enables Myo2 to localize to the division site (Motegi et al., 2004). Myo2 localizes to the division site in both F-actin-dependent and -independent manners (Figure 3D; Wu et al., 2003; Motegi et al., 2004; Takaine et al., 2014). Mid1 and its downstream component Rng2 are essential for the F-actin-independent localization of Myo2 or its C-terminal targeting domain to the cytokinetic nodes prior to actin ring assembly (Laporte et al., 2011; Padmanabhan et al., 2011; Takaine et al., 2014). This may involve the interactions between Mid1, Rng2, and the localization domain of Myo2, which were detected by co-immunoprecipitation experiments, but not detected by direct protein-binding assays using purified proteins or their fragments (Motegi et al., 2004; Padmanabhan et al., 2011; Takaine et al., 2014). Mid1 disappears from the division site at the time of actin ring assembly (Padmanabhan et al., 2011; Takaine et al., 2014). Afterward, Rng2 is required for the maintenance of F-actin-independent Myo2 at the division site (Takaine et al., 2014). This Rng2-dependent targeting of Myo2 and the potential interaction between Rng2 and the targeting domain of Myo2 is remarkably similar to the Iqg1-mediated Myo1 localization during cytokinesis in budding yeast (Fang et al., 2010).

Myp2 is recruited to the division site during anaphase B, after the actin ring assembly (Wu et al., 2003). Despite its later arrival than Myo2 at the division site, the Myp2 ring constricts slightly earlier than the Myo2 ring (Mcdonald et al., 2017; Okada et al., 2019). This might account for the geometric separation of the myosin-II isoforms in the constricting ring in which the Myp2 ring is located inside of the Myo2 ring (Laplante et al., 2015; Mcdonald et al., 2017). Myp2 localization to the division site completely depends on F-actin (Figure 3D; Wu et al., 2003) and requires the collective contributions of its head domain, the first CC region, and the NHR (residues 1–1,615) (Figure 1; Takaine et al., 2015; Okada et al., 2019). In interphase cells, Myp2 forms “spots” next to the nucleus at high temperature (Wu et al., 2006). The formation of the spot depends on the second CC region of the Myp2 tail, and these spots may be associated with the γ-tubulin complex (Okada et al., 2019), although the function and regulation of these spots remain unknown.

Myo2, the formin Cdc12, and actin filaments in the contractile ring are dynamic, turning over in 30–60 s (Pelham and Chang, 2002; Clifford et al., 2008; Yonetani et al., 2008; Stachowiak et al., 2014). A number of *in silico* and *in vivo* analyses indicates that proper turnover is required for tension generation and uniform constriction of the ring components (Stachowiak et al., 2014; Oelz et al., 2015; Thiyagarajan et al., 2017; Alonso-Matilla et al., 2019; Cheffings et al., 2019). In contrast to Myo2, Myp2 is largely immobile during cytokinesis (Wloka et al., 2013; Takaine et al., 2015; Okada et al., 2019). Similar to Myo1 in *S. cerevisiae* (Wloka et al., 2013), truncation of a C-terminal region in Myp2 abolishes its immobility (Okada et al., 2019). The same C-terminal region of Myp2 also renders Myo2 less dynamic when it is fused to the tail of Myo2 (Okada et al., 2019). Thus, the C-terminal region of Myp2 is critical for its immobility, and, importantly, fully accounts for the contribution of Myp2 to the rate of AMR constriction (Okada et al., 2019). How the immobility of Myp2 is established remains unknown. As the C-terminal region of a myosin-II is generally required for its self-assembly, we hypothesize that the loss of the C-terminal region in Myp2 might prevent its assembly into a higher-order structure such as the kernel of a Myp2 cluster, as proposed previously (Takaine et al., 2015). Alternatively, but not mutually exclusively, the loss of the C-terminal region in Myp2 could abolish its interaction with other proteins or subcellular structures such as the post-anaphase array (PAA), a microtubule array formed at the division site during cytokinesis (Samejima et al., 2010). Strikingly, Myp2 lacking the C-terminal fragment or the 2nd CC region is able to localize to the division site with the same efficiency as the full-length Myp2 and fully mediates its role in high-salt response (Bezanilla and Pollard, 2000; Takaine et al., 2015; Okada et al., 2019). Thus, distinct regions of Myp2 are responsible for its localization and dynamics during the cell cycle.

### The Functions of Myo2 and Myp2 in Cytokinesis

#### Roles of Myo2 and Myp2 in Cytokinesis Under Normal Growth Conditions

Myo2 plays an essential role in cell viability and cytokinesis (Kitayama et al., 1997; May et al., 1997), and this role requires both its head and tail domains (Lord et al., 2005). In contrast, Myp2 plays a fine-tuning role in cytokinesis under normal growth conditions and this role can be visualized only when the Myo2 function is compromised (Laplante et al., 2015; Palani et al., 2017; Okada et al., 2019). This role must also require both the head and tail domains of Myp2, as both domains are essential for its localization to the division site (Takaine et al., 2015; Okada et al., 2019).

The AMR in permeabilized fission yeast cells or “cell ghosts” constricted in an ATP- and myosin-II-dependent manner (Mishra et al., 2013). This constriction was completely blocked by blebbistatin, a specific inhibitor of myosin-IIs, suggesting that the collective motor activities of Myo2 and Myp2 are essential for the AMR constriction (Mishra et al., 2013). This is supported by the analyses of motor-domain mutations in both Myo2 and Myp2 (Mishra et al., 2013; Takaine et al., 2015; Pollard and O’Shaughnessy, 2019), as *myo2-E1* and *myp2-R694C* display additive effects on ring constriction (Takaine et al., 2015). Using this cell-ghost system, the rates of ring constriction in *myp2Δ* and *myo2-E1* cell were determined to be approximately 2/3 and 1/3 of the rate in WT cells, respectively (Mishra et al., 2013). However, the rings in the cell ghosts of the *myp2Δ myo2-E1* double mutant were frequently fragmented or deformed and failed to undergo ATP-dependent constriction, suggesting that the motor activity of Myo2 is required for the assembly and/or maintenance of the AMR (Mishra et al., 2013). When spheroplasts (i.e., the cells after the removal of cell walls) and micropipette aspiration were used to measure membrane tension, the average ring tensions of the WT, *myp2Δ*, and *myo2-E1* cells were ∼640, 400, and 220 pN, respectively (Pollard and O’Shaughnessy, 2019). Again, the ring tensions in *myp2Δ* and *myo2-E1* cells were about 2/3 and ∼1/3 of the ring tension in WT cells, respectively (Pollard and O’Shaughnessy, 2019). Thus, two independent studies came to the same conclusion that both Myo2 and Myp2 contribute to the ring constriction in fission yeast, with Myo2 playing a more prominent role (Pollard and O’Shaughnessy, 2019). This conclusion is further supported by the measurements of the relative contributions of the myosin-II isoforms to ring constriction in intact cells, which indicates that Myp2 accounts for ∼30% of the rate of constriction (Okada et al., 2019). Strikingly, Myp2 lacking the last ∼200 residues (i.e., the C-terminal portion of the 2nd CC region) does not affect the accumulation kinetics of Myp2 at the division site, but reduces the rate of constriction to the same level as *myp2* null does (Okada et al., 2019). This observation suggests that the last portion of Myp2 tail might be required for the appropriate organization of Myp2 in the contractile ring in order for its motor domain to function in constriction. Despite significant progress in experimental and modeling studies, how Myo2 and Myp2 act in concert to drive AMR assembly and constriction *in vivo* remains unclear (Pollard and O’Shaughnessy, 2019).

AMR constriction is thought to depend on the binding and sliding of actin filaments by myosin-II. The relative contributions of the binding and sliding to AMR assembly and constriction were determined through the genetic and biochemical analysis of the motor-deficient allele *myo2-E1* and its suppressor (Palani et al., 2017). The *myo2-E1* is temperature-sensitive for growth and division. The mutated amino acid (G345R) in the *myo2-E1* allele induces a steric hindrance that causes deficiencies in ATPase activity and actin-filament binding *in vitro* (Balasubramanian et al., 1998; Lord and Pollard, 2004; Stark et al., 2013; Palani et al., 2017, 2018). The fact that the *myo2-E1* cells can grow and divide reasonably well at the permissive temperature whereas *myo2* null is inviable suggests that a motor-independent function of Myo2 likely exists. A genetic screen for the suppressors of the temperature-sensitive growth of the *myo2-E1* cells has led to the identification of an intragenic suppressor (Sup1) that contains two amino-acid changes, Q640H and F641I (Palani et al., 2017). Surprisingly, the gene product of *myo2-E1-Sup1* can bind, but cannot slide, actin filaments *in vitro*, and the isolated AMRs from the *myo2-E1-Sup1* cells fail to undergo ATP-dependent contraction (Palani et al., 2017). Thus, *myo2-E1-Sup1* defines an actin translocation-defective allele (Palani et al., 2017). While the *myo2-E1-Sup1* cells can grow and divide at the high temperature that is restrictive for *myo2-E1*, the AMR assembly is delayed, and the rate of ring constriction is decreased (Palani et al., 2017). These observations suggest that the binding of Myo2 to actin filaments is sufficient for AMR assembly and constriction *in vivo*, and the motor or filament-sliding activity of Myo2 is required for efficiency (Palani et al., 2017). Likewise, mutational analysis of the Myp2 motor domain suggests that the ability to bind actin filaments is more important than the motor activity for its function (Takaine et al., 2015). Given that the actin translocation activity of myosin-II is dispensable for cytokinesis in mammalian COS-7 cells (Ma et al., 2012), the essential role of myosin-II in cytokinesis appears to be dictated by its ability to bind and cross-link the actin filaments whereas the motor activity makes the processes of ring assembly and constriction more efficient. This is fundamentally the same as the headless Myo1 in budding yeast, which can direct AMR assembly and constriction, although with decreased efficiency (Fang et al., 2010). In the absence of the motor activity, the AMR constriction is perhaps driven, at least in part, by cell cycle-triggered actin depolymerization, as suggested previously (Mendes Pinto et al., 2012).

Because the AMR is incapable of generating sufficient tension to overcome the turgor pressure and cell wall stiffness (Proctor et al., 2012; Stachowiak et al., 2014), the contractility of the ring is unlikely to be the direct driving force for furrow ingression. Alternatively, the rate of PS synthesis determines the rate of constriction (Figure 3E; Stachowiak et al., 2014). The PS in *S. pombe* consists of linear β-1,3-glucan synthesized by Bgs1/Cps1, hereafter Bgs1 (Cortes et al., 2007). Conditional inactivation of PS synthesis in the temperature-sensitive *cps1-191* mutant does not prevent AMR assembly but attenuates its constriction (Liu et al., 1999; Dundon and Pollard, 2020). Thus, PS formation is required for efficient AMR constriction and furrow ingression. Bgs1 appears to anchor to the AMR (Arasada and Pollard, 2014; Davidson et al., 2016; Sethi et al., 2016; Martin-Garcia et al., 2018). Moreover, Bgs1 and its nascent product strictly follow the constriction of the AMR structure (Mulvihill and Hyams, 2003a; Cortes et al., 2007; Okada et al., 2019). Thus, the AMR might play a pivotal role in cytokinesis by acting as “scaffold” or “compass” that guides PS formation, whereas the contractile force produced by the AMR might play a supportive role in cytokinesis by regulating Bgs1 activity (Thiyagarajan et al., 2015) or by generating a tiny space between the cell wall and the PM at the leading edge of the ingressing furrow for efficient PS synthesis.

The mechanisms underlying the roles of Myo2 and Myp2 in guiding PS formation remain poorly understood. The depletion of Myo2 leads to haphazard deposition of PS materials at the division site, suggesting that Myo2 plays a critical role in guiding PS formation (Okada et al., 2019). A previous analysis indicates that a defect in AMR assembly can be suppressed by a block in PS formation, suggesting that the arrival of Bgs1 and PS formation before the completion of AMR assembly compromises later aspects of cytokinesis (Huang et al., 2008). In support of this conclusion, when the AMR assembly is compromised (e.g., *myo2-E1* at the restrictive temperature), Bgs1 localizes to the misoriented myosin-II cables, instead of a ring structure, at the medial region and starts synthesizing the PS materials by following the myosin cables (Figure 3E; Okada et al., 2019). Thus, myosin-II can guide PS formation even when it is not incorporated into a ring structure. However, timely and correct AMR assembly is essential for “centripetal” PS formation (Okada et al., 2019). It is worth noting that the guiding role of Myo2 does not require its motor activity, as the actin translocation-defective allele of *myo2*^+^ (i.e., *myo2-E1-Sup1*) can largely accomplish cytokinesis and guide PS formation (Palani et al., 2017). This is strikingly similar to the headless Myo1, which does the same in budding yeast (Fang et al., 2010).

Myp2 may also play a role in guiding PS formation. Myp2 starts to constrict earlier than Myo2, and is internal to Myo2 during ring constriction (Laplante et al., 2015; Okada et al., 2019). Deletion of *myp2*^+^ delays the onset of constriction and decreases the rate of constriction (Figure 3E; Laplante et al., 2015; Okada et al., 2019). Myp2 is also required for AMR assembly and maintenance when the motor activity of Myo2 is inactivated (e.g., in the *myo2-E1* or *myo2-E1-Sup1* mutant) (Takaine et al., 2015; Palani et al., 2017). Furthermore, Myp2 is immobile (Wloka et al., 2013; Takaine et al., 2015; Okada et al., 2019), similar to Myo1 in budding yeast that acts as scaffold during cytokinesis (Wloka et al., 2013). Taken together, it is tempting to speculate that Myp2 might play a role in guiding PS formation by reinforcing the AMR structure and controlling its timely and fast constriction at the leading edge of the ingressing membrane.

F-actin is also involved in guiding the PS formation, but is only required during the early phase of ring constriction. Association of Bgs1 with the myosin-II and the activation of PS formation can occur in a F-actin-independent manner (Okada et al., 2019). However, F-actin is required for the confinement of Bgs1 at the membrane edge during the early phase of ring constriction (Ramos et al., 2019), but is dispensable for Bgs1 localization and cytokinesis during the second half of ring constriction (Proctor et al., 2012; Okada et al., 2019). Further analysis indicates that F-actin is required for maintaining Myo2 at the division site during the first half of ring constriction. Afterward, Myo2 can maintain its localization at the division site in the absence of F-actin (Okada et al., 2019). Thus, F-actin is indirectly involved in guiding PS formation by concentrating and organizing Myo2 at the division site during the early phase of ring constriction (Okada et al., 2019).

#### The Interplay of Myo2 and Myp2 in Cytokinesis During Stress Response

Deletion of *myp2*^+^, but not the *myo2-E1* mutant, leads to loss of viability and defects in cytokinesis in the presence of high salt (Bezanilla and Pollard, 2000; Fujita et al., 2002; Baker et al., 2016), suggesting that Myp2 plays a unique role in coping with environmental stresses. Myosin-II behavior in stress response has been under-studied. Recent time-lapse imaging analysis, coupled with microfluidic devices, indicates different responses of Myo2 and Myp2 upon high salt stress (Okada et al., 2019). Importantly, Myp2 is required for the dynamic disassembly and stability of the AMR during stress response (Figure 3F; Okada et al., 2019), but the underlying mechanism remains unknown. The cells lacking calcineurin, a conserved Ca^2+^- and calmodulin-dependent protein phosphatase, show a similar phenotype to *myp2Δ* cells such as defective cytokinesis and high salt sensitivity (Sugiura et al., 1998). A constitutive-active form of calcineurin suppresses salt sensitivity of the *myp2Δ* cells, suggesting that Myp2 might be required for ion homeostasis and/or activation of calcineurin (Fujita et al., 2002). Myp2 interacts with Ste20 (not the homologue of the PAK Ste20 in budding yeast), the rictor subunit of the TOR complex 2 (TORC2), that is required for cell viability in high salt (Matsuo et al., 2007; Baker et al., 2016). Myp2 and Ste20 are interdependent for their localization, and Ste20 is required for efficient cytokinesis (Baker et al., 2016). Given the importance of TORC2 in general stress responses, Myp2 may be required for stress adaptation of cytokinesis via TORC2-mediated pathways.

### Major Unanswered Questions Regarding Myo2 and Myp2 in Fission Yeast

Despite significant progress made in recent years, many key aspects of the structure, function, and regulation of myosin-IIs in fission yeast remain unclear. The organizational patterns of Myo2 and Myp2 at the division site must be determined, as they are essential for understanding the mechanisms of AMR assembly and tension generation. The motor-dependent and -independent functions of myosin-IIs in cytokinesis require further investigation, as this is crucial for understanding the relative contributions of the binding, sliding, and disassembly of actin filaments to the rate of ring constriction. It also remains largely unknown how PS formation, the primary driving force of ingression, is regulated by the AMR. More specifically, it remains unclear whether and how the AMR interacts with Bgs1 to achieve uniform ingression during cytokinesis.

It is also a mystery how Myo2 and Myp2 act in concert to drive AMR assembly and constriction under normal and stressed conditions. While the structure and organization of Myo2 at the division site has been extensively analyzed, even the basic questions regarding Myp2 remain unanswered: is Myp2 a single-headed myosin, as suggested by the *in vitro* study (Bezanilla and Pollard, 2000)? How is its immobility established during cytokinesis? How does it generate tension or act as a scaffold to facilitate furrow ingression? Of particular interest is how Myp2 is regulated to render the division machinery resistant to various stresses. Finally, the existence of proline residues in the middle portion of the myosin tail is conserved among fungal species (Mulvihill and Hyams, 2003b), but the function and mechanism of this NHR is not well understood. In budding yeast, this region, also called TD2, is required for Iqg1-mediated Myo1 localization at the division site during cytokinesis (Fang et al., 2010). The NHR in Myp2 is also required for its localization to the division site (Takaine et al., 2015), but the underlying mechanism remains unknown.

## Myh9, Myh10, and Myh14: The Three Non-Muscle Myosin-II Heavy Chains in Mammalian Cells

Humans diverged from the budding yeast and fission yeast almost a billion years ago (Hoffman et al., 2015; Liu et al., 2017). Along the way, the human genome has evolved to encode three NM-II heavy chains Myh9, Myh10, and Myh14 that define the NM-II isoforms IIA, IIB, and IIC, respectively (Figure 1; Conti and Adelstein, 2008; Vicente-Manzanares et al., 2009; Heissler and Sellers, 2015). Each heavy chain binds an ELC (Mlc3nm or Mlc1sa) encoded by either *MYL6* or *MYL6B*, and an RLC (Mlc2, Mlc2B, or Mlc2A) encoded by *MYL9*, *MYL12A*, or *MYL12B* (Heissler and Sellers, 2015). Similar to budding yeast and fission yeast, the ELC in mammalian cells also binds to IQGAPs, but the function of this binding remains unknown (Weissbach et al., 1998; Atcheson et al., 2011; Pathmanathan et al., 2011). Different cell types usually express two or three of the isoforms (Golomb et al., 2004; Ma et al., 2010; Pecci et al., 2018). Deletions of different NM-II isoforms in mice cause distinct phenotypes. Deletion of NM-IIA causes embryonic lethality at day 6.5 and defects in cell adhesion and endoderm formation (Conti et al., 2004). Deletion of NM-IIB causes embryonic lethality at day 14.5 and defects in heart and brain functions including cardiac myocyte cytokinesis (Ma et al., 2010). Deletion of NM-IIC alone does not cause lethality or obvious defects, but, when combined with decreased level of NM-IIB, causes lethality and karyokinesis in cardiac myocytes by affecting microtubule dynamics (Tullio et al., 1997; Ma et al., 2010). Studies using the mouse model and different cell lines indicate that NM-IIs play critical roles in tissue morphogenesis, cell migration, cytokinesis, and exocytosis (Conti and Adelstein, 2008; Vicente-Manzanares et al., 2009; Shutova and Svitkina, 2018). Despite decades of research, the answers to the major questions of how different isoforms are elaborately regulated to achieve an appropriate ratio and how these isoforms act in concert to carry out specific functions in a given cell type just began to emerge. Here, we will focus on the roles of the NM-II isoforms in cytokinesis.

### The Structure and Assembly of the NM-IIA, -IIB, and -IIC

The NM-II heavy chains Myh9, Myh10, and Myh14 in mammalian cells share a similar domain organization as the myosin-II heavy chains in budding yeast and fission yeast, a globular head domain with actin-binding and ATPase activities, IQ1 and IQ2 motifs where an ELC and an RLC bind, respectively, and a rod-shaped tail that is made of CCs, followed by a small NHR in each isoform (Figure 1). Similar to the myosin-IIs in budding yeast (Luo et al., 2004) and fission yeast (D’souza et al., 2001), deletion of the IQ2 motif in NM-IIA does not appear to compromise its ability to localize to the division site or to drive cytokinesis (Beach and Egelhoff, 2009). The biochemical property of each NM-II isoform, its monomer structure and its assembly into bipolar filaments, and the regulation of its filament assembly by RLC and heavy chain phosphorylation as well as by interacting proteins have been studied extensively *in vitro* as well as in the context of cell adhesion, polarization, and migration (Vicente-Manzanares et al., 2009; Shutova and Svitkina, 2018). In addition, specific mutations in the NM-IIA gene, which cause *MYH9*-related diseases, have been analyzed in terms of their impact on IIA structure and functions in developmental processes as well as in the pathogenesis of the relevant diseases (Vicente-Manzanares et al., 2009; Pecci et al., 2018). These subjects have been reviewed comprehensively in several recent articles (Vicente-Manzanares et al., 2009; Pecci et al., 2018; Shutova and Svitkina, 2018). Here, we only describe some salient features of the NM-IIA, -IIB, and -IIC in relation to their roles in cytokinesis.

**FIGURE 4:**
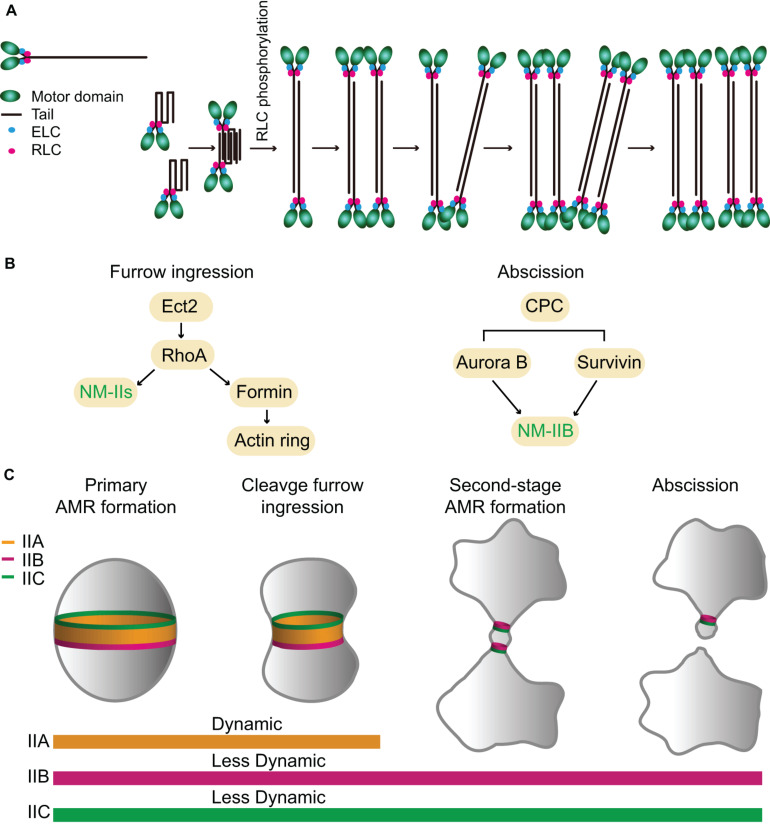
Assembly, localization, dynamics, and function of NM-IIA, -IIB, and -IIC during cytokinesis in mammalian cells. **(A)** A model for NM-IIA assembly. Modified from Fenix et al. (2016); Beach et al. (2017), and Liu et al. (2018). **(B)** Targeting mechanisms of NM-IIs during furrow ingression and abscission. **(C)** Localization patterns and dynamics of different NM-II isoforms during furrow ingression and abscission. See text for details.

The structure and organization of these NM-IIs were studied using both native myosin and sf9 expressed proteins (Pollard et al., 1974; Niederman and Pollard, 1975; Billington et al., 2013; Liu et al., 2018). Like Myo1 in budding yeast and Myo2 in fission yeast, the monomers of all three NM-II isoforms are heterohexamers that consist of two heavy chains, two ELCs, and two RLCs (Figure 4A; Billington et al., 2013). These monomers can further polymerize into bipolar filaments of ∼300 nm in length *in vitro*, although with isoform-specific features. The bare zone length of IIC is much longer than that of IIA or IIB, but the width of IIC filaments is smaller than that of IIA or IIB (Billington et al., 2013; Liu et al., 2018). IIA and IIB filaments each contain ∼30 myosin monomers whereas IIC filaments contain ∼16 myosin monomers (Billington et al., 2013; Melli et al., 2018). *In vitro* analysis suggests that NM-II filament assembly starts with folded monomers that interact with each other to form folded antiparallel dimers, which assemble into folded antiparallel tetramers that are unfolded via RLC phosphorylation to form a bipolar tetramer (Liu et al., 2018). The unfolded bipolar tetramers associate with each other to form bipolar filaments (Figure 4A; Liu et al., 2018). *In vivo* analyses indicate that NM-II bipolar filaments assemble into “stacks,” presumably, to increase their capacity for force production (Figure 4A; Verkhovsky et al., 1995, 1999; Fenix et al., 2016; Fenix and Burnette, 2018). Time-lapse analysis by super-resolution microscopy suggests that the assembly of IIA filament stacks follows the same pathway or the “expansion model” at the leading edge of a motile cell (U2-OS) or at the cleavage furrow of a dividing cell (HeLa): the bipolar filaments first recruit other bipolar filaments to become thicker, and then a part of the thick filament bundle branches out to recruit more bipolar filaments, and, eventually, form a IIA filament stack (Figure 4A; Fenix et al., 2016; Fenix and Burnette, 2018). Further live-cell imaging by super-resolution microscopy of mouse fibroblasts carrying a knock-in EGFP-NM-IIA indicates that IIA filaments nucleate at the cell periphery, grow, split, expand, and stack up as they move deeper into the cell to form large-scale actomyosin structures and this process depends on actin dynamics (Beach et al., 2017). Similar conclusions on myosin filament stacking and its dependency on actin organization have been reached by an independent study (Hu et al., 2017; Titus, 2017). It remains unknown whether IIB and IIC form stacks in the same way.

The stack-like organization of the IIA filaments at the division site in HeLa cells (Fenix et al., 2016) shows some similarities and differences to the organization of the NM-II filaments at the division site in sea urchin embryos (Henson et al., 2017). Both appear to organize into arrays of myosin filaments along the circumference of the division site, which is consistent with the classic “purse-string model” of cytokinesis (Schroeder, 1972; Rappaport, 1996). However, the registration of the head and tail domains of the bipolar filament assemblages in sea urchin embryos is not as ordered as that in HeLa cells (Fenix et al., 2016; Henson et al., 2017). In addition, NM-II appears to form discrete clusters at the division site during early stage of cytokinesis in sea urchin embryos (Henson et al., 2017). These clusters coalesce into myosin filament arrays over time, a process that resembles the node-to-ring assembly process in fission yeast. Whether the assemblies of mammalian NM-IIs go through a similar process during cytokinesis remains unknown.

### Localization and Dynamics of the NM-IIA, -IIB, and -IIC During Cytokinesis

The expression levels of the three NM-II isoforms vary greatly in HeLa-Kyoto cells. IIA is expressed 16-fold and 135-fold higher than IIB and IIC, respectively (Maliga et al., 2013). During cytokinesis, they localize to the division site for different periods of time. IIA localizes to the division site only during furrow ingression and disappears at the midbody stage, whereas IIB and IIC localize to the division site during both the furrowing and the abscission stages (Wang et al., 2019). The endogenous IIB and IIC localize to the constriction sites in HeLa-Kyoto cells and A549 (human lung tumor cells), respectively, before abscission (Jana et al., 2006; Wang et al., 2019). During furrow ingression, the localization of IIA and IIB at the division site depends on local RhoA activation by its guanine-nucleotide-exchange factor Ect2 whose localization is, in turn, dictated by the centraspindlin complex (Figure 4B; Yuce et al., 2005; Piekny and Glotzer, 2008; Zhou and Wang, 2008; Frenette et al., 2012). Active RhoA also controls actin ring assembly via formins (Figure 4B; Watanabe et al., 2008). Thus, RhoA is required for AMR assembly in mammalian cells (Figure 4B; Basant and Glotzer, 2018; Verma et al., 2019). The chromosomal passenger complex (CPC), including its components Aurora B and survivin, appears to regulate IIB localization during the abscission stage (Figure 4B; Kondo et al., 2013; Babkoff et al., 2019). Similar to Myo1 in budding yeast (Fang et al., 2010), the central region of the IIA tail (residues 834–1,632) is required for its localization to the division site (Figure 1), but its tethering factor at the division site remains unknown (Beach and Egelhoff, 2009). Because the septin-binding protein Bni5 and the IQGAP Iqg1 in budding yeast (Fang et al., 2010) and the anillin-like protein Mid1 and the IQGAP Rng2 in fission yeast (Motegi et al., 2004; Laporte et al., 2011; Padmanabhan et al., 2011; Takaine et al., 2014) are required for the targeting of myosin-II heavy chain to the division site during the cell cycle, similar proteins such as anillin and/or IQGAPs might be involved in the localization of NM-IIA to the division site in mammalian cells. The C-terminal region of IIA (residues 1,633–1,960) is able to localize to the division site only by interacting with the endogenous IIA or IIB in the cell (Beach and Egelhoff, 2009), which is strikingly similar to the behavior of the C-terminal “putative assembly domain” of Myo1 in budding yeast (Fang et al., 2010; Wloka et al., 2013). How RhoA and other factors control the localization of different isoforms at the division site during different stages of cytokinesis requires further investigation.

FRAP analysis indicates that IIA is highly dynamic, whereas IIB and IIC are less dynamic during cytokinesis (Figure 4C; Kondo et al., 2011; Wang et al., 2019). The differential turnover of IIA and IIB at the division site is similar to that of IIA and IIB during cell migration (Sandquist and Means, 2008). This isoform-specific behavior appears to be dictated by a C-terminal region in their respective tails (Sandquist and Means, 2008). As both IIA and IIB assemble into bipolar filaments of similar size and shape *in vitro* (Billington et al., 2013), it remains unclear how their turnover is differentially regulated *in vivo*.

### The Functions of NM-IIA, -IIB, and -IIC in Cytokinesis

Mammalian cytokinesis is divided into two stages: furrow ingression and abscission. During furrow ingression, the diameter of the cell at the division site shrinks from its initial size (∼10–20 μm for most cell types) to the diameter of the intercellular bridge (ICB) between the daughter cells at the time of the midbody formation (1.5–2 μm) (Mullins and Biesele, 1973, 1977). The prevalent idea is that furrow ingression is driven by the contractile ring, which disassembles at the midbody stage, whereas abscission is carried out by the endosomal sorting complex required for transport-III (ESCRT-III) (Elia et al., 2011; Guizetti et al., 2011; Agromayor and Martin-Serrano, 2013). However, the ESCRT-III complex is known to act on membrane tubes with much smaller dimension (∼100–300 nm in diameter) (Henne et al., 2012; Agromayor and Martin-Serrano, 2013; Chiaruttini et al., 2015; Alonso et al., 2016). Thus, the ICB must be further thinned significantly in order to permit the ESCRT-III to fulfill its role in abscission.

#### Differential Requirements of NM-IIs in Furrow Ingression and Abscission

During furrow ingression, all three isoforms localize to the division site and play redundant or overlapping roles, as depletion of IIA or IIB alone or in combination did not block furrowing in HeLa-Kyoto cells, but the addition of 25 μM blebbistatin, a specific inhibitor of myosin-IIs (Straight et al., 2003; Zhang et al., 2017), completely arrested cytokinesis at the furrowing stage (Wang et al., 2019). Strikingly, the addition of a lower dosage of blebbistatin (7.5 μM) allowed furrowing, but significantly delayed or blocked abscission (Wang et al., 2019). Thus, the motor activity of NM-II plays a critical role in abscission, not just at the furrowing stage (Wang et al., 2019). Importantly, GFP-tagged IIB and IIC, but not IIA, still localize to the division site during the abscission stage (Figure 4C; Wang et al., 2019). Further analysis indicates that the endogenous IIB (but not IIA), F-actin, and Sept9 localize to the constriction sites during the early midbody stage whereas the ESCRT-III components arrive there only during the late midbody stage (Wang et al., 2019). Strikingly, the addition of the lower dosage of blebbistatin completely abolished the constriction site formation at either side of the midbody and prevented the normal localization of the above-mentioned factors at the constriction sites (Wang et al., 2019). Together, these observations suggest that NM-IIs are not only required for furrow ingression, but also for abscission. Furthermore, these observations suggest that different NM-II isoforms play differential roles in abscission, with IIB (and IIC) playing a local and direct role in the formation of the constriction sites and IIA playing a more global and indirect role in abscission by exerting cortical tension to drive the daughter cells apart (Wang et al., 2019).

It has become increasingly clear that there are two spatiotemporally separated actomyosin rings that operate during mammalian cytokinesis (Figure 4C; Wang et al., 2019). The primary AMR drives furrow ingression and it requires all NM-II isoforms whereas the second-stage AMRs drive the formation of the constriction sites for the subsequent abscission (Wang et al., 2019). These second-stage rings consist of similar components as those in the primary AMR, e.g., RhoA, formins, F-actin, anillin, and septins, with the exception that the second-stage rings contain IIB and likely IIC, but not IIA (Jana et al., 2006; Hu et al., 2012; Renshaw et al., 2014; Dema et al., 2018; Karasmanis et al., 2019; Wang et al., 2019). Thus, the isoforms of NM-IIs define a difference between the primary and second-stage AMRs, which presumably account for their distinct roles in furrow ingression and abscission during cytokinesis. It is noteworthy that the IIB-based second-stage AMR is remarkably similar to the Myo1-based AMR in budding yeast in terms of size, composition, and myosin-II dynamics.

#### Distinct Biochemical Properties of NM-IIs May Account for Their Distinct Roles in Furrow Ingression and Abscission

Several recent studies indicate that IIA generates cortical tension and drives furrow ingression during cytokinesis, whereas IIB controls cortical stability and cytokinetic fidelity, perhaps by attenuating the IIA-dependent rate of rapid ingression (Yamamoto et al., 2019; Taneja et al., 2020). These observations are consistent with the distinct biochemical properties of IIA and IIB, i.e., IIA is “designed” for rapid motility whereas IIB is best for maintaining static tension (Golomb et al., 2004; Kovacs et al., 2007; Zhang et al., 2017; Melli et al., 2018). These observations are also consistent with the differential localization patterns and dynamics of IIA and IIB at the division site (Wang et al., 2019). Besides cytokinesis, IIA and IIB also play distinct roles in cell migration and cell–cell adhesion (Sandquist and Means, 2008; Shutova et al., 2014; Shutova and Svitkina, 2018; Heuze et al., 2019). In migrating cells, IIA is preferentially localized to the leading edge where it displays rapid turnover. In contrast, IIB is incorporated into actomyosin structures at the rear end and displays much less turnover than IIA does (Sandquist and Means, 2008; Shutova et al., 2014; Shutova and Svitkina, 2018). During the early stage of adherent junction formation in epithelial cells, IIA binds to the actin bundles that are in parallel to the adherent junctions (Efimova and Svitkina, 2018; Heuze et al., 2019) and functions to elongate the junctions by producing a contractile force (Heuze et al., 2019). In contrast, IIB localizes to the branched actin network that might serve as a cross linker of the junctional actin network to maintain the rigidity of the structure (Heuze et al., 2019). These functions of IIA and IIB are also consistent with the general notion that IIA is specialized for force production whereas IIB is specialized for maintaining tension. Together, these observations suggest that the distinct biochemical properties of NM-IIs may account for their distinct roles in furrow ingression and abscission during cytokinesis.

### Major Questions Regarding the Roles of Different Isoforms in Mammalian Cytokinesis

While the NM-II isoforms in mammalian cells are best studied for their *in vitro* behaviors such as in motility and filament assembly, their mechanisms of action for their *in vivo* roles such as in cytokinesis remain poorly understood. There are many outstanding questions. For example, it remains unknown how and why different isoforms are regulated to achieve a cell type-specific ratio to drive furrow ingression and abscission. What is the architecture of the primary and second-stage AMRs? Is the myosin-II organization in the contractile ring nearly identical to that in stress fibers, as suggested by a recent analysis of IIA at the division site in HeLa cells (Fenix et al., 2016)? It is also unknown whether different NM-II isoforms are co-assembled into the same bipolar filaments, as seen in the LLC-Pk1 cells (Beach et al., 2014), or the co-assembly merely represents an intermediate stage for sorting IIA and IIB into distinct filaments at distinct locations (Beach et al., 2014; Shutova et al., 2014). Are IIA and IIB filaments spatially segregated at the division site as Myo2 and Myp2 in fission yeast or in other spatial patterns (e.g., the edge vs. the center of the cleavage furrow)? How are the primary and second-stage AMRs assembled during the cell cycle? Does the furrow ingression depend on the motor activity, as suggested by the blebbistatin experiments (Straight et al., 2003; Wang et al., 2019) or is it mainly dictated by the binding of myosin heads to the actin filaments not their sliding on the filaments, as suggested by the behavior of an actin translocation-defective mutation in IIB in COS-7 cells (Ma et al., 2012)? Both motor-dependent and-independent activities of myosin-IIs are known to play a role in cytokinesis in budding yeast (Lord et al., 2005; Fang et al., 2010) and fission yeast (Mishra et al., 2013; Palani et al., 2017).

## Conclusion and Perspective

Several important lessons are learned through the comparative analysis of the roles of the NM-II isoforms in cytokinesis in budding yeast, fission yeast, and mammalian cells. First, the commonalities regarding the roles of the NM-IIs in cytokinesis in different systems seem to be far more significant than their differences. The NM-IIs in all three systems assemble into a functional AMR in anaphase and constrict to drive furrow ingression. Their differences in the timing of arrival at the division site, which seems to be over-emphasized in the literature, may have nothing to do with cytokinesis or are only involved in fine-tuning the division process. For example, Myo1 in budding yeast localizes to the division site in the absence of an actin ring from bud emergence (G/S) to the onset of anaphase (Bi et al., 1998; Lippincott and Li, 1998). This portion of Myo1 localization is responsible for its role in the retrograde flow of actin cables (Huckaba et al., 2006), and erasing Myo1 localization at the division site before anaphase by deleting Bni5, the linker protein between the septin hourglass and Myo1, does not cause any obvious defects in cytokinesis (Fang et al., 2010). Similarly, Myo2 in fission yeast localizes to the cytokinetic nodes in the absence of an actin ring upon entry into mitosis (Wu et al., 2003, 2006). These nodes are presumably involved in linking nuclear position (usually at the mid-point of the cell) to the division site (Sohrmann et al., 1996; Bahler et al., 1998; Paoletti and Chang, 2000). However, these nodes are not essential for cytokinesis, as abolishing the nodes by deleting the anillin-like protein Mid1 only affects the location and efficiency of the AMR assembly, and once the AMR is assembled, cytokinesis can occur normally (Huang et al., 2008). Thus, starting in anaphase when the AMR assembly occurs in budding yeast, fission yeast, and mammalian cells, the roles of NM-IIs in cytokinesis appear to be very similar in all three systems. The pre-anaphase NM-II structures at the division site in the two yeast species are presumably evolved to fit the lifestyle of each organism, i.e., budding and binary fission.

Second, it is remarkable that, in all three systems, cytokinesis can largely occur in the absence of the motor activity of the NM-IIs or when the motor activity is severely compromised, but the execution of cytokinesis requires the binding of the NM-IIs to the actin filaments. This is demonstrated abundantly clear by the behaviors of the headless AMR in budding yeast (Lord et al., 2005; Fang et al., 2010), and of the actin translocation-defective NM-IIs in fission yeast (Palani et al., 2017) and mammalian cells (Ma et al., 2012). Thus, the assembly of an AMR and the tension generated by the myosin–actin interaction in the ring are critical for cytokinesis whereas the motor activity merely makes the process more efficient. This raises an important question: how does furrow ingression occur in the absence of the motor activity? One possibility is that the cell cycle-triggered actin and myosin filament disassembly drives furrow ingression in all three systems. Additionally, in the case of the budding yeast and fission yeast, the AMR in the absence of the motor activity can still guide PS formation, which, in turn, could drive furrow ingression (Fang et al., 2010; Proctor et al., 2012; Palani et al., 2017; Okada et al., 2019).

Finally, why do the budding yeast, fission yeast, and mammalian cells possess one, two, and three NM-II isoforms, respectively? There are a number of possibilities. First, it may be related to the dimension of the division site in different systems (Fang et al., 2010). The division sites in budding yeast, fission yeast, and the mammalian cells are 1, 3.5, and > 10 μm in diameter, respectively (Mitchison, 1957; Bi et al., 1998; Lippincott and Li, 1998; Straight et al., 2003; Carvalho et al., 2009). In order to accomplish cytokinesis in a defined window of time during the cell cycle, the fission yeast and mammalian cells may have acquired a fast-turnover NM-II (Myo2 in fission yeast and IIA in mammalian cells) that is specialized in rapid contraction whereas the more “ancient” NM-IIs are probably the slow-turnover ones (e.g., Myo1 in budding yeast, Myp2 in fission yeast, and IIB and IIC in mammalian cells) that are kept to maintain the cortical stability of the division site (Okada et al., 2019; Wang et al., 2019). These slow-turnover NM-IIs may scaffold PS formation in budding yeast (Fang et al., 2010; Wloka et al., 2013) and fission yeast (Proctor et al., 2012; Palani et al., 2017; Okada et al., 2019) or the assembly of the abscission machinery in mammalian cells (Wang et al., 2019). Second, more than one NM-II isoform in fission yeast and mammalian cells might be evolved to enable cytokinesis under environmental stresses such as the fission yeast Myp2 in dealing with survival under high-salt stress at the low temperature (Bezanilla and Pollard, 2000; Okada et al., 2019). Another possibility is that the additional isoforms of NM-II are evolved to expand the repertoire of their functions. For example, IIA and IIB play distinct roles not only in cytokinesis, but also in cell migration, junctional complex formation, exocytosis, and many other processes (Sandquist and Means, 2008; Shutova et al., 2014; Milberg et al., 2017; Efimova and Svitkina, 2018; Shutova and Svitkina, 2018; Heuze et al., 2019). Regardless of the possibilities, the core questions of how different NM-II isoforms are elaborately regulated to achieve a cell type-specific ratio and how different isoforms are assembled into a specific architecture to drive cytokinesis in different cell types require extensive investigation.

## Author Contributions

KW and HO wrote the initial draft. EB revised the manuscript. All authors read and approved the final manuscript.

## Conflict of Interest

The authors declare that the research was conducted in the absence of any commercial or financial relationships that could be construed as a potential conflict of interest.
